# Ceramic Thermal Insulator Based on Diatomite Obtained by Starch Consolidation Casting

**DOI:** 10.3390/ma16114028

**Published:** 2023-05-28

**Authors:** Cinthya Alvarado, Hernán Alvarado-Quintana, Raúl Siche

**Affiliations:** 1 Carrera de Ingeniería Civil, Facultad de Ingeniería, Universidad Privada del Norte, Trujillo 13011, Peru; 2 Departamento de Ingeniería de Materiales, Facultad de Ingeniería, Universidad Nacional de Trujillo, Trujillo 13011, Peru; halvarado@unitru.edu.pe; 3 Facultad de Ciencias Agropecuarias, Universidad Nacional de Trujillo, Trujillo 13011, Peru; rsiche@unitru.edu.pe

**Keywords:** diatomite, starch, starch consolidation, porous ceramics, diametral compression, thermal insulator, microstructure, viscosity, insulation panel, thermal conductivity

## Abstract

Researchers are continuously seeking to develop new materials to protect against inclement weather and thus optimize energy efficiency in housing. This research aimed to determine the influence of corn starch percentage on the physicomechanical and microstructural properties of a diatomite-based porous ceramic. The starch consolidation casting technique was applied to fabricate a diatomite-based thermal insulating ceramic with hierarchical porosity. Diatomite mixtures with 0%, 10%, 20%, 30%, and 40% starch were consolidated. The results show that starch content significantly influences apparent porosity, and this, in turn, influences several parameters, such as thermal conductivity, diametral compressive strength, microstructure, and water absorption of diatomite-based ceramics. The porous ceramic processed by the starch consolidation casting method corresponding to the mixture of diatomite with 30% starch obtained the best properties, with a thermal conductivity of 0.0984 W/m·K, an apparent porosity of 57.88%, a water absorption of 58.45%, and a diametral compressive strength of 35.18 kg/cm^2^ (3.45 MPa). Our results reveal that the diatomite-based ceramic thermal insulator obtained by starch consolidation is effective for use on roofs to improve thermal comfort in dwellings located in cold regions.

## 1. Introduction

Humans have always sought out materials to protect themselves from inclement weather. Environmental pollution and energy-related challenges have both experienced progressive worsening in recent years [1]. According to [2], the energy use of buildings accounts for around 40% of worldwide energy consumption and more than 30% of global CO_2_ emissions. As mentioned in [3], the tension between the supply and demand of energy is growing because of the depletion of traditional fossil fuel supplies and the inefficient use of already existing energy sources. Enhancing energy efficiency and creating new energy sources have emerged as crucial strategies for resolving this paradox. As [4] states, most of a building’s energy consumption depends on the outdoor climate. Many cities experience extremely high or low temperatures, with a direct impact on the exterior walls of buildings; thus, thermal comfort must be maintained inside the home.

Porous ceramics play an important role in our daily lives; they are used in applications such as thermal insulation and barriers, acoustic absorption, vibration attenuation, energy storage, and fluid filtration [5]. As argued by [6], due to benefits such as low density, low thermal conductivity, high specific surface area, stable chemical characteristics, heat and corrosion resistance, and other qualities, porous ceramics are frequently employed in filtration, adsorption, heat insulation, catalyst support, and other applications. Porous ceramics are created using a variety of techniques, including the replica route [7], sacrificial template technology [8], the direct foaming method [9], starch consolidation casting (SCC) [10], gel casting [11], foam gel casting [12] and freeze-drying technology [13].

There are currently two main areas of concentration for insulating material research and development. The goal of encouraging the use of environmentally friendly building materials and products is becoming increasingly important, which, in turn, is encouraging the development of insulation materials with a lower environmental impact throughout their entire life cycle, favoring those that are quickly renewable, derived from plants or animals, potentially recyclable, non-toxic, and have low energy production methods [14].

Diatomite is a naturally occurring sedimentary rock made of the siliceous remains of fossilized diatoms [15]. In addition to its amorphous silica content, diatomite frequently contains carbonate and clay minerals, quartz, and feldspars. These minerals are readily accessible and inexpensive in large quantities [16]. Due to their chemical inertness, exceptional mechanical qualities, vast surface area, excellent water absorption, very porous structure between 80 and 90% [17], and very low pore size ranging from 100 nm to 100 μm, diatomaceous earths have attracted interest from a variety of study sectors [15]. Because they include amorphous silica, are light, have fine pores, are porous, and have low thermal conductivity (0.05–0.10 W/m·K), they can be utilized as filters, as catalyst carriers, and as heat, cold, and sound insulators [15]. Diatomite is used in the building industry to make lightweight bricks and ceramics and as a lightweight aggregate in mortar and concrete [18]. The use of lightweight aggregates, which have strong thermal insulation characteristics because of their porous nature, may be a method for increasing the insulation ability of concrete components [19]. Diatomite has also been used as a substitute for fly ash in foamed geopolymers, which are used as thermal insulators [20].

One of the commonly used pore-forming substances in ceramic technology is starch. The ceramic forming technology that uses starch is known as starch consolidation casting, in which starch additionally acts as a binder [10]. A high-molecular-weight polymer generated from plants, starch is found in many crops, including potato tubers, maize cobs, and cassava roots [21]. It is made up of two sugars, amylose and amylopectin [22]. For the food, paper, pharmaceutical, and chemical sectors, starch provides a low-cost, biologically renewable source of feedstock [21]. This natural biopolymer is easily burned out during burning without leaving any residues in the finished ceramic body due to its chemical makeup [23].

Thermal insulators are highly resistant to the passage of heat, reducing heat transfer; i.e., they insulate against cold or heat, resulting in savings in energy use by increasing their thermal resistance [24]. There are organic and inorganic thermal insulation panels on the market; however, the organic ones have the disadvantage of being flammable, the fibrous inorganic ones are harmful to health, and the particulate inorganic ones densify when sintered at high temperatures, so new processing methods that incorporate controlled porosity are required [25]. However, nowadays there are ceramic foams that do not densify at high temperatures and have lower conductivities [26].

For this reason, this research seeks to develop a diatomite-based material that has a hierarchical pore structure and can be used as an efficient thermal insulator to improve thermal comfort in houses located in cold regions. This research work is justified because it develops a new material for obtaining an efficient thermal insulator to help address frost and cold. In this way, it optimizes the conservation of internal heat in the house by reducing thermal conductivity, which will improve people’s thermal comfort and quality of life. Based on the above, we formulate the following question: What is the influence of the percentage of corn starch on the microstructure, apparent porosity, water absorption, diametral compressive strength, and thermal conductivity of a diatomite-based ceramic?

## 2. Materials and Methods

### 2.1. Materials

The main material used in this research was diatomite obtained from the company Silicis Perú, whose deposits are in Piura, Peru. This diatomite has the following characteristics: density 2.06 g/cm^3^, BET surface area 12.99 m^2^/g, and particle size less than 45 μm (325 mesh), as guaranteed by the supplier. The chemical composition of diatomite was obtained by X-ray fluorescence in the Bizalab laboratory in Lima, Peru. The identification of the crystalline phases present in the diatomite prior to processing was carried out using a Rigaku Mini-flex 600 X-ray diffractometer and QualX 2.0 software. The operating conditions to obtain the diffractometry were a Kα line of copper, wavelength 1.54 A°, with a step of 0.02°, a sweep between 10 and 80° at a speed of 2° per minute, a voltage of 40 kV, and a current intensity of 15 milliamperes.

The material used as a pore-forming agent was commercial corn starch. Its morphology and chemical composition were determined with a Tescan Vega 3 scanning electron microscope with an Oxford EDS attached. Corn starch is a granular polysaccharide composed of two glucose polymers, amylose (amorphous and soluble) and amylopectin (crystalline and insoluble); the amylose/amylopectin ratio determines the different degrees of crystallinity of the grain [27].

### 2.2. Experimental Design

The experimental design was unifactorial, having as an independent variable the percentage of starch in the diatomite–starch mixture, which was investigated at 5 levels: 0%, 10%, 20%, 30%, and 40% starch. The dependent variables were apparent porosity (%), water absorption (%), and diametral compressive strength (kg/cm^2^). All tests were performed in triplicate, except for diametral compressive strength, which was performed in quintuplicate.

### 2.3. Experimental Procedure

**Figure 1:**
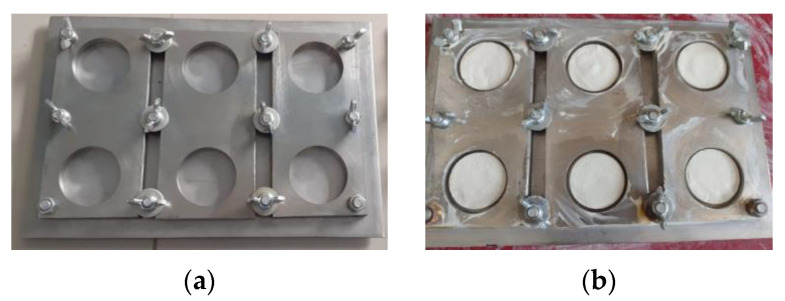
(**a**) Stainless steel molds; (**b**) Samples formed by the SCC method.

The obtaining of the raw materials and their characterization are described in Section 2.1. Stainless steel molds were made to produce 55 mm-diameter by 10 mm-high disks (Figure 1), resistant to drying and gelling temperatures in the oven. A preliminary study of the rheology of the slips of diatomite–starch mixtures prepared at 42% solids was then carried out. For this purpose, an Anton-Paar Viscosimeter model Visco QC 100 L was used, and deflocculation curves were constructed to determine the amount of sodium silicate (deflocculant) to be added to the slips so that they remain stable and have the appropriate viscosity to be easily strained. In this study, one more point was added (50% starch) to see if the optimum amount of sodium silicate to be added tended to be maintained. Once the amount of deflocculant was determined, the starch consolidation casting method formed the tablets. The slips were prepared by stirring the water with the deflocculant at 300 rpm and gradually adding the diatomite–starch solid mixtures until a 42% solids slip was obtained, with a total stirring time of 1 h. The slips were strained on a vibrating table regulated at a vibration frequency of 10 rpm for 5 min. Then, they were transferred in their entirety and molded in an oven to gel at a temperature of 65 °C for 2 h. They were then dried in an oven for 24 h at a temperature of 110 °C. They were left to cool at room temperature, and all the tablets were unmolded.

The demolded tablets were then placed in an electric furnace to be calcined at 600 °C for 3 h to eliminate organics. Then, they were sintered at 1000 °C in the same electric furnace for 1 h.

Apparent porosity and water absorption were calculated using ASTM C373-18. This standard test method is based on Archimedes’ principle. The samples were oven-dried at 110 °C. The dry mass *D* was determined using a 0.01 g precision balance. The samples were then placed in a container with distilled water and boiled for 5 h. After this, all specimens were left immersed in water for an additional 24 h. The suspended mass *S* was determined on a 0.01 g precision hydrostatic balance. Finally, the saturated mass *M* was determined by blotting it superficially with a moistened cloth on a 0.01 g precision balance. The external volume *V* of each specimen was calculated as the difference of *M* minus *S*.

The following equation was used to compute the apparent porosity *P*:

(1) $$P=\left[\frac{\left(M-D\right)}{V}\right]\times 100$$

The water absorption *A* was calculated using the following equation:

(2) $$A=\left[\frac{\left(M-D\right)}{D}\right]\times 100$$

Diametral compressive strength was determined according to ASTM D-3967-16. By compressing two lines in opposite directions, this test establishes a disc’s maximum tensile strength *σ_t_*. A Humboldt HM-3000 compression press with a 50 kN load cell was used at a speed of 0.1 mm/min. The maximum breaking load *P*, diameter *d*, and thickness *t* of each specimen were determined. The diametral compressive strength was calculated using the following formula:

(3) $$\sigma_{t}=\frac{2P}{\pi td}$$

Thermal conductivities were measured using a system implemented according to ASTM C-177-19 based on Fourier’s law. This standard applies the protected hot plate method, which is a steady-state measurement technique that uses the electrical power output of a hot plate with guided heat conduction to evaluate the thermal conductivity of a material. This measurement system was calibrated with an expanded polystyrene (EPS) plate of known thermal conductivity. InstaCal’s software version 6.73 was used for data acquisition, and TracerDAQ software version 2.3.4.0 was used for plotting the graph, which was used to verify that the temperature measurements were taken in a steady state.

The Tescan Vega 3 XMU scanning electron microscope was used to obtain the microstructure that allowed us to differentiate the porosities obtained at the different scales.

Finally, the variation in the formation of crystalline phases in the unsintered diatomite with the diatomite sintered at 1000 °C was determined. Likewise, they were compared with the best mixture of diatomite and starch sintered at the same temperature.

## 3. Results

### 3.1. Characterization of Used Materials

The chemical composition of diatomite is shown in Table 1. The results showed that the main component of diatomite is silica (SiO_2_).

**Figure 2:**
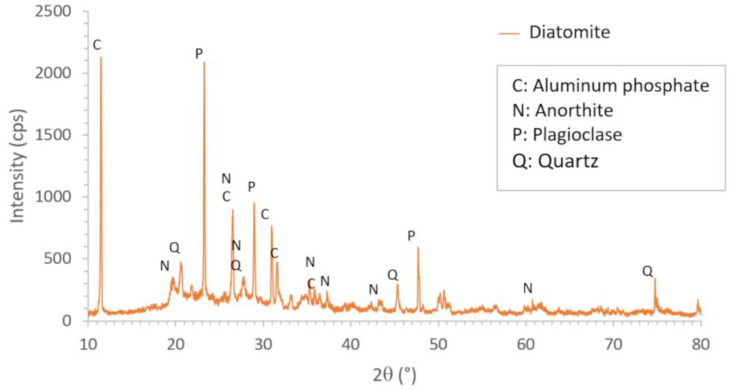
X-ray diffractogram of diatomite.

Figure 2 shows the X-ray diffractogram of diatomite. It shows the peaks corresponding to the crystalline phases, whose characteristics are shown in Table 2. A considerable amount of amorphous phase is also observed in the lower part of the diagram (noise), leading us to determine that the degree of crystallinity of this material was 22.95%.

**Figure 3:**
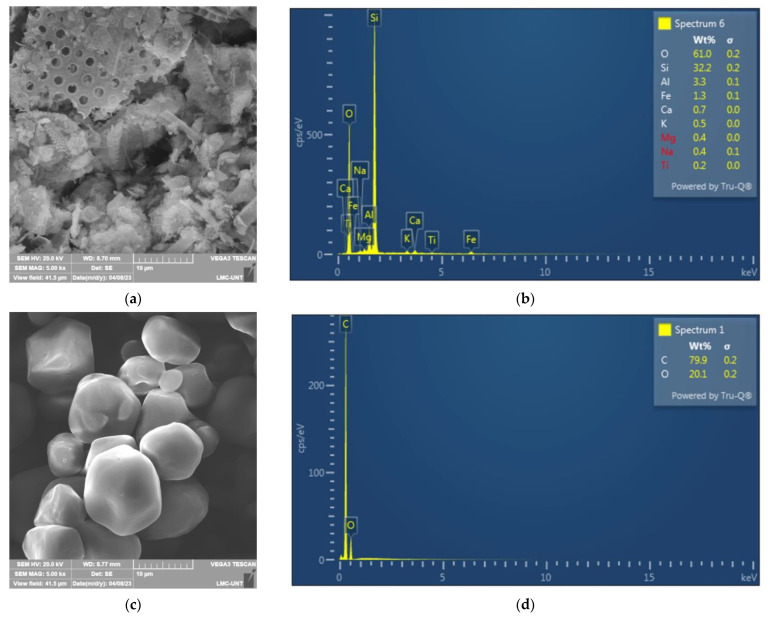
(**a**) SEM micrograph of diatomite; (**b**) EDS of diatomite; (**c**) SEM micrograph of corn starch; (**d**) EDS of corn starch.

Figure 3a shows a micrograph of diatomite at 5000×. Figure 3b shows its energy dispersive spectroscopy (EDS) analysis. Figure 3c shows a SEM micrograph of corn starch at 5000× showing a particle size of about 10 μm. Figure 3d shows its EDS analysis.

Finally, to stabilize the slips, which are aqueous suspensions of diatomite–starch, liquid sodium silicate with a SiO_2_/Na_2_O ratio of 3.35 was used as a deflocculant, in an amount determined by its flocculation curve.

### 3.2. Rheological Study to Establish the Quantity of Deflocculant

**Figure 4:**
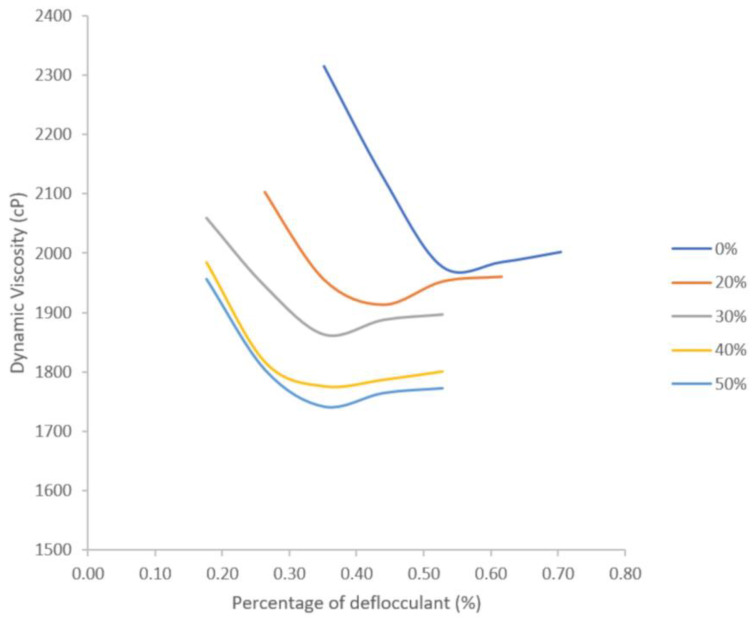
Deflocculation curves of diatomite–starch slips.

Figure 4 shows the dynamic viscosities (cP) as a function of the percentage of sodium silicate added as a deflocculant for the investigated slips with different corn starch contents. On the one hand, it is observed that as the percentage of starch in the diatomite–starch solid mixture increases, the viscosity of the slip decreases. On the other hand, it is observed that as the percentage of sodium silicate added as deflocculant increases, the viscosity decreases continuously until reaching a value for which the viscosity no longer decreases but, on the contrary, begins to increase slightly.

### 3.3. Apparent Porosity

**Figure 5:**
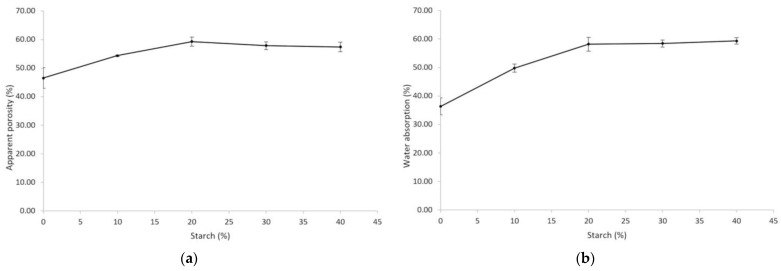

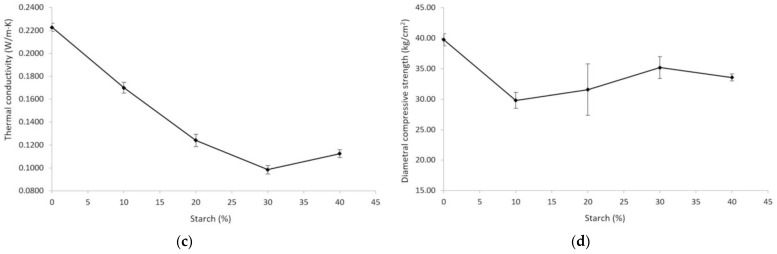
(**a**) Apparent porosity as a function of starch percentage; (**b**) Water absorption as a function of starch percentage; (**c**) Thermal conductivity as a function of starch percentage; (**d**) Diametrical compressive strength as a function of starch percentage.

Figure 5a presents the apparent porosity (%) as a function of the percentage of starch added to the diatomite–starch solid mixture. It can be stated that as the starch percentage increases, the apparent porosity grows in a decreasing manner, i.e., the increase is smaller each time the independent variable is increased. One of the reasons for adding starch to the diatomite was so that it would form porosity on a different scale than that of diatomite alone.

### 3.4. Water Absorption

Figure 5b shows the water absorption (%) as a function of the percentage of starch added to the diatomite–starch solid mixture. In the same way as for apparent porosity, it can be stated that as the percentage of starch increases, the water absorption grows in a decreasing way. This can be attributed to the fact that water absorption depends on the pore area that is exposed, and therefore, it will have the same behavior as the one observed for apparent porosity. Traditional porous ceramics are mostly classified by their ability to absorb water.

### 3.5. Thermal Conductivity

Figure 5c shows the thermal conductivity (W/m·K) as a function of the percentage of starch added to the diatomite–starch solid mixture. It can be stated that as the percentage of starch increases, the thermal conductivity decreases up to a minimum threshold, after which it rises slightly again.

### 3.6. Diametral Compressive Strength

Figure 5d shows the diametral compressive strength as a function of the percentage of starch added to the diatomite–starch solid mixture. In general, it can be stated that diatomite without starch has higher strength than diatomite–starch mixtures. Likewise, it can be stated that in diatomite–starch mixtures, as the percentage of starch increases, the diametral compressive strength increases until reaching a maximum, at which point the strength begins to decrease.

### 3.7. Microstructures Obtained by SEM

**Figure 6:**
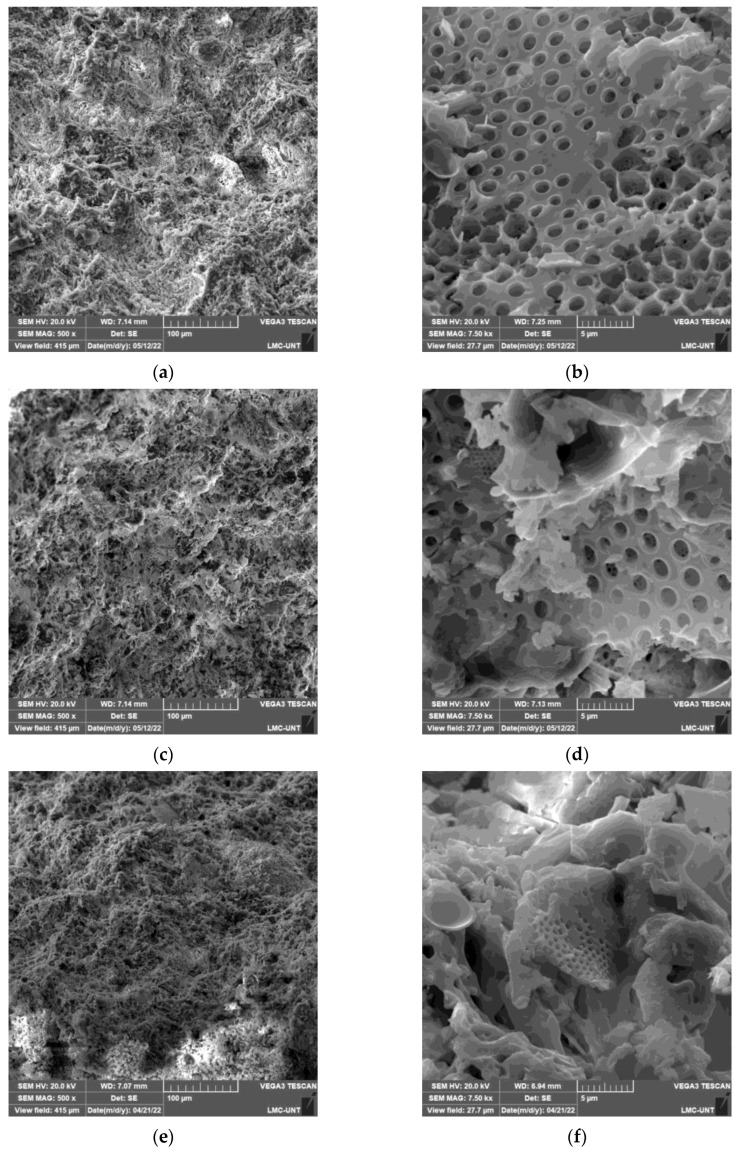

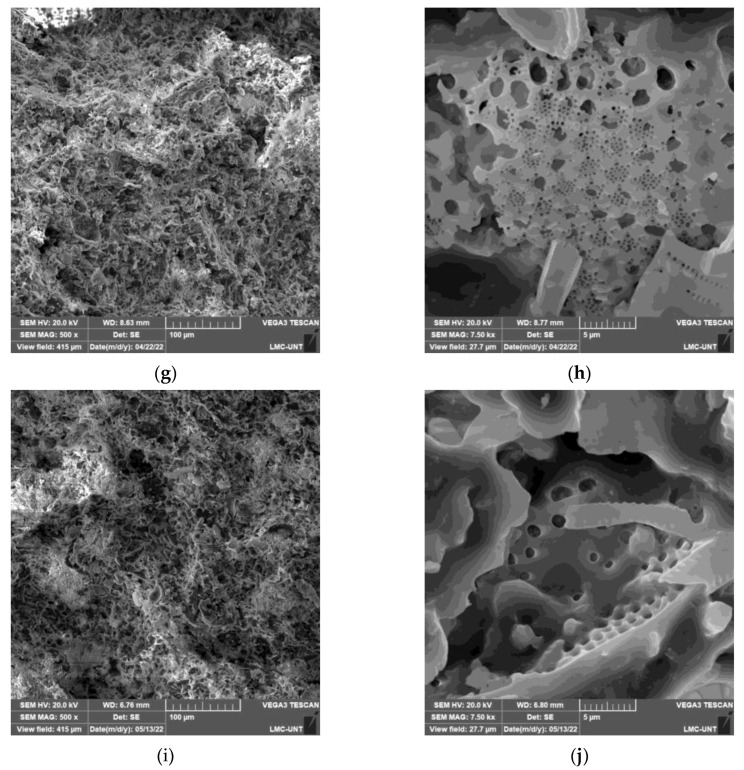
SEM microstructures of the porous ceramics studied: (**a**) Sample a1: diatomite, 0% starch at 500×; (**b**) Sample a2: diatomite, 0% starch at 7500×; (**c**) Sample b1: diatomite, 10% starch at 500×; (**d**) Sample b2: diatomite, 10% starch at 7500×; (**e**) Sample c1: diatomite, 20% starch at 500×; (**f**) Sample c2: diatomite, 20% starch at 7500×; (**g**) Sample d1: diatomite, 30% starch at 500×; (**h**) Sample d2: diatomite, 30% starch at 7500×; (**i**) Sample e1: diatomite, 40% starch at 500×; (**j**) Sample e2: diatomite, 40% starch at 7500×.

Figure 6 shows the SEM micrographs of diatomite (a1 and a2) and diatomite–starch (b1, b2, c1, c2, d1, d2, e1, and e2). In diatomite without starch, it can be observed in (a2) that it has only porosity in the microscale and very few pores (a1) in the mesoscale.

On the other hand, in the diatomite–starch materials (b1, b2, c1, c2, d1, d2, e1, and e2), it is observed that mesopores increasingly form with greater amounts of added starch. These formed pores are of the order of 10 to 15 microns, as observed in the microphotographs at 500 magnification. In the 7500-magnification micrograph e2, a large pore occupies almost the entire microphotograph.

### 3.8. XRD of the Sintered Samples

**Figure 7:**
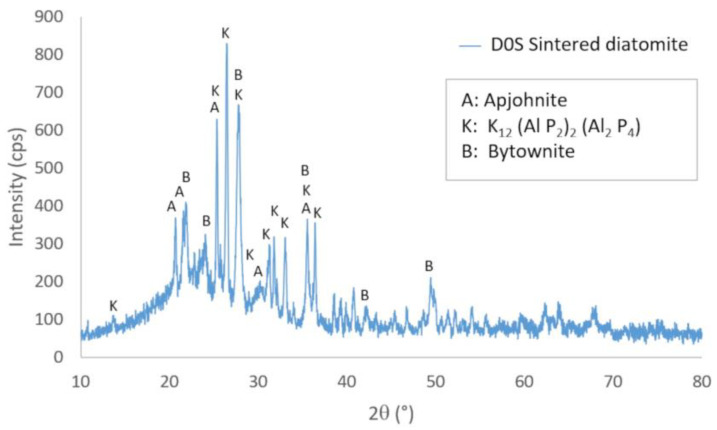
X-ray diffractogram of diatomite sintered at 1000 °C (D0S).

Figure 7 shows the X-ray diffractogram of diatomite sintered at 1000 °C (D0S). It shows the peaks corresponding to the crystalline phases, whose characteristics are shown in Table 3. A considerable amount of amorphous phase is also observed, determining that its degree of crystallinity is 29.31%. This is because, at high temperatures, crystalline phases at room temperature are destroyed and transformed into new crystalline phases, which need time to crystallize.

**Figure 8:**
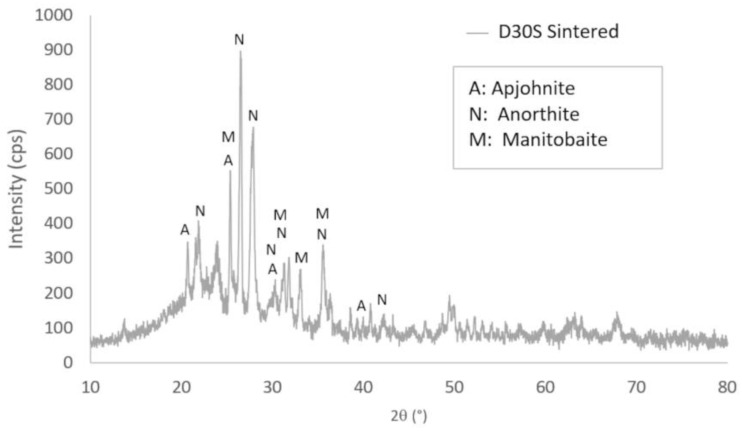
X-ray diffractogram of the mixture D30S sintered at 1000 °C.

Figure 8 shows the X-ray diffractogram of the mixture that gave the best results, which was diatomite with 30% starch (D30S) sintered at 1000 °C. It displays the peaks associated with the crystalline phases, whose traits are displayed in Table 4. Because the crystalline phases at ambient temperature are destroyed at high temperatures and changed into new crystalline phases that need time to solidify, a significant amount of amorphous phase is also seen, with a degree of crystallinity of 26.54%.

**Figure 9:**
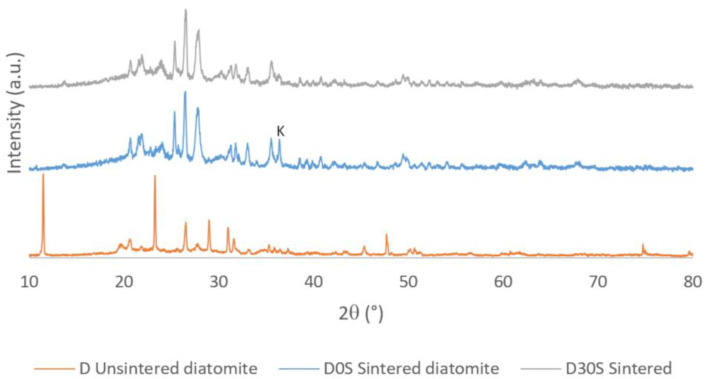
Superimposed XRD to identify phase transformations at high temperatures.

Finally, Figure 9 shows the three X-ray diffractograms superimposed to identify the phase transformations that occur when the samples are subjected to the 1000 °C temperature. All the crystalline phases of diatomite at room temperature are destroyed at high temperatures, and new crystalline phases are created because of the interaction of these phases. These transformations require time and higher temperatures to complete and form stable phases at high temperatures. It should be noted that the phase called K is a new phase of the sintered diatomite that tends to transform into another phase due to the presence of starch residues that accelerate its transformation.

## 4. Discussion

From the deflocculation curves carried out in the rheological study (Figure 4), it was established that for all the slips containing starch, 0.40% of sodium silicate should be used, while for the slips without starch, 0.55% of sodium silicate should be used; these values are optimum for the slips to have the lowest viscosity to facilitate the filling of the molds. The sodium silicate has the function of modifying the surface charge of the diatomite particles by negatively charging them in such a way that they repel each other, preventing them from coming together and reaching the necessary weight to settle and stabilizing the suspension; however, this also decreases the viscosity of the slip, making it more fluid and easier to fill all the interstices of the mold. These results are in agreement with the results of Lee et al. [28].

Regarding the behavior observed in Figure 5a of the decreasing growth of apparent porosity as the starch percentage increases, it can be explained that when small amounts of starch are added, the gel formed is uniformly distributed in layers of minimal thickness that facilitate its burning, obtaining a greater number of small pores with a greater amount of pore area. On the other hand, with higher amounts of added starch, the gel formed results in thicker layers that are not uniformly distributed and are segregated into certain islands, requiring more energy and more time to eliminate them and forming fewer pores and, therefore, a smaller pore area. When the starch content increased from 0 wt.% to 40 wt.%, the apparent porosity increased from 46.54 wt.% to 59.28 wt.%. These findings are consistent with those of other authors, as reported in their studies [6,13,29]. This is corroborated by analyzing the SEM microphotographs in Figure 6.

Analyzing the water absorption curve (Figure 5b), we can state that it has the same behavior as the apparent porosity curve (Figure 5a), that is, the water absorption also presents decreasing growth as the starch percentage increases. This is explained in the same way: at small amounts of added starch, the gel constituted is uniformly distributed in layers of minimal thickness that facilitate its burning, obtaining a greater number of small pores and thus generating a greater amount of pore area and a greater water absorption capacity. On the other hand, at higher quantities of added starch, the gel formed results in thicker layers that are not uniformly distributed and are segregated into certain islands, requiring more energy and more time to eliminate them and forming fewer pores; therefore, there is less pore area and thus less water absorption capacity. When the starch content increased from 0 wt.% to 40 wt.%, the water absorption increased from 36.35 wt.% to 59.35 wt.%. These results are slightly lower than those obtained by Ho et al. [29].

The behavior observed in the thermal conductivity curve as a function of the percentage of starch added (Figure 5c) is explained by the fact that the main mechanism of heat conduction in ceramic materials is the phonon. A phonon is a quantized mode of vibration (a vibrational wave) that takes place in the atomic lattices of a solid, transporting heat through atoms from zones of higher to lower vibration. According to the Peierls-Boltzmann transport equation, the thermal conductivity at the atomic level is proportional to the mean free path, which is the path in which the phonon travels undisturbed [30]. This means that as the amount of starch in the mixture increases, more discontinuities (pores) are produced, and therefore, there is less thermal conductivity. This occurs until the optimum pore ratio is reached, after which the starch begins to segregate and form larger and more distant pores; consequently, the mean free path is greater, and the thermal conductivity rises. Diatomite consolidated with 30% starch gives a thermal conductivity of 0.0984 W/m·K. These results coincide with and, in some cases, improve on the thermal insulation found by authors who have used other techniques [6,13,20,29,31].

The behavior of diametrical compressive strength (Figure 5d) is explained considering the double function of starch: on the one hand, it is a pore-former, and as such, it is a defect-former; as resistance is sensitive to the presence of defects (pores), in general, diatomite–starch mixtures have lower resistances. The diametral compression test is an indirect tension test, that is, it generates a tensile state, which opens the crack oriented parallel to the application of the load. In tension, the resistance is inversely proportional to the size of the largest flaw. According to this, it would be expected that by increasing the starch, the resistance decreases, but we observe that the resistance increases up to a maximum; this is explained by the other function that starch serves, which is to be a binder [32,33]. Increasing the starch increases the amount of gel formed, which serves to better bind the particles and facilitate their rearrangement, obtaining smaller interparticle spaces and greater densification after sintering, thus achieving better mechanical strength. The maximum diametral compressive strength found with 30% consolidated starch was 35.18 kg/cm^2^, which is equivalent to 3.45 MPa. These findings are consistent with those of other authors, as reported in their studies [6,13,29,31].

From the SEM micrograph in Figure 6, there is a large amount of closed porosity at nanometer sizes, and as more starch is incorporated, these closed pores increase in quantity up to 30% starch. A high degree of open pores will result in convective contributions, while closed pores will reduce convective contributions in addition to giving the possibility of trapping a low-conductivity gas in the pores. It is highly probable that starch combustion will result in the presence of CO_2_ in closed pores, which has a lower thermal conductivity than atmospheric air [34]. Therefore, we recommend further study to determine the exact percentage of closed pores formed.

## 5. Conclusions

This research work presents the successful synthesis of a diatomite-based porous ceramic, obtained by the starch consolidation method, to be used as thermal insulation in roofs to improve thermal comfort in cold areas. The main findings are summarized as follows:

(1) “Influence of starch content”: Starch content significantly influences the apparent porosity, and this, in turn, influences several parameters, such as thermal conductivity, diametral compressive strength, microstructure, and water absorption of the diatomite-based ceramic. As starch content increases, apparent porosity and water absorption increase, while thermal conductivity and diametral compressive strength decrease. This is important because the lower the thermal conductivity of a material, the better it is as a thermal insulator, and the greater the thermal comfort inside the house.
(2) “Optimal diatomite–starch mixture”: The optimal mixture was diatomite with 30% starch. With this mixture, ceramic tiles can be manufactured to provide thermal insulation for residential roofs in cold regions. This mixture had a thermal conductivity of 0.0984 W/m·K, an apparent porosity of 57.88%, a water absorption of 58.45%, and a diametral compressive strength of 35.18 kg/cm^2^ (3.45 MPa). These values of thermal conductivity and thermal resistance are adequate for a thermal insulating material.
(3) “Thermal insulator with hierarchical porosity”: A cellular ceramic with hierarchical porosity at the nanoscale, microscale, and mesoscale was obtained, making it suitable for application as an effective thermal insulator. It is important for thermal insulators to have pores of different sizes, because this will make it more difficult for heat to flow through them.

In summary, our findings reveal that the developed thermal insulator is suitable to be used to improve thermal comfort in houses located in cold regions. To improve its mechanical strength, it is recommended that in future work, kaolin be incorporated as the third component of the solid mixture to act as the main binder, leaving starch only as a pore-forming agent. In addition, a more exhaustive study of the closed porosity is recommended, because the carbon dioxide generated by the starch combustion could also influence the thermal conductivity by being trapped in the closed pores and having a thermal conductivity lower than that of air.

## Figures and Tables

**Table 1:** X-ray fluorescence analysis of diatomite.

Chemical Compound	SiO_2_	Al_2_O_3_	CaO	SO_3_	Fe_2_O_3_	MgO	Na_2_O	K_2_O	P_2_O_5_	Cl	TiO_2_	SrO	V_2_O_5_	Cr_2_O_3_	ZnO	LOI
wt.%	59.96	10.69	4.99	4.05	4.02	2.86	2.01	1.45	1.21	1.06	0.33	0.09	0.04	0.02	0.02	7.2

**Table 2:** Crystalline phases of unsintered diatomite obtained by XRD.

Compound Name	Formula	Crystal System	Percentage
C: Aluminum phosphate	Al P O_4_	Triclinic	21.6%
N: Anorthite	Ca (Al_2_ Si_2_ O_8_)	Triclinic	33.0%
P: Plagioclase	Al_3_._88_ Ca_0_._88_ H_2_ Na_0_._12_ O_12_ Si_2_._12_	Monoclinic	36.3%
Q: Quartz	SiO_2_	Triclinic	9.1%

**Table 3:** Crystalline phases of sintered diatomite (D0S) obtained by XRD.

Compound Name	Formula	Crystal System	Percentage
A: Apjohnite	Al_2_ Fe_0_._02_ H_44_ Mg_0_._28_ Mn_0_._64_ O_38_ S_4_ Zn_0_._06_	Monoclinic	46.3%
K: K_12_ (Al P_2_)_2_ (Al_2_ P_4_)	Al_4_ K_12_ P_8_	Triclinic	26.5%
B: Bytownite	Al_7_._76_ Ca_3_._44_ Na_0_._56_ O_32_ Si_8_._24_	Triclinic	27.2%

**Table 4:** Crystalline phases of the sintered D30S mixture obtained by XRD.

Compound Name	Formula	Crystal System	Percentage
A: Apjohnite	Al_2_ Fe_0_._02_ H_44_ Mg_0_._28_ Mn_0_._64_ O_38_ S_4_ Zn_0_._06_	Monoclinic	51.2%
N: Anorthite	Al_2_ Ca O_8_ Si_2_	Triclinic	39.0%
M: Manitobaite	Al_6_._56_ Ca_2_._44_ Fe_12_ Mg_0_._44_ Mn_12_._2_ Na_15_._13_ O_120_ P_30_	Monoclinic	9.8%

## Data Availability

The data presented in this study are available on request from the corresponding author.
